# Vaccine effectiveness against hospitalization among adolescent and pediatric SARS-CoV-2 cases between May 2021 and January 2022 in Ontario, Canada: A retrospective cohort study

**DOI:** 10.1371/journal.pone.0283715

**Published:** 2023-03-31

**Authors:** Alison E. Simmons, Afia Amoako, Alicia A. Grima, Kiera R. Murison, Sarah A. Buchan, David N. Fisman, Ashleigh R. Tuite

**Affiliations:** 1 Dalla Lana School of Public Health, University of Toronto, Toronto, Ontario, Canada; 2 Public Health Ontario, Toronto, Ontario, Canada; 3 Centre for Immunization Readiness, Public Health Agency of Canada, Ottawa, Ontario, Canada; Wingate University, UNITED STATES

## Abstract

**Background:**

Vaccines against SARS-CoV-2 have been shown to reduce risk of infection as well as severe disease among those with breakthrough infection in adults. The latter effect is particularly important as immune evasion by Omicron variants appears to have made vaccines less effective at preventing infection. Therefore, we aimed to quantify the protection conferred by mRNA vaccination against hospitalization due to SARS-CoV-2 in adolescent and pediatric populations.

**Methods:**

We retrospectively created a cohort of reported SARS-CoV-2 case records from Ontario’s Public Health Case and Contact Management Solution among those aged 4 to 17 linked to vaccination records from the COVaxON database on January 19, 2022. We used multivariable logistic regression to estimate the association between vaccination and hospitalization among SARS-CoV-2 cases prior to and during the emergence of Omicron.

**Results:**

We included 62 hospitalized and 27,674 non-hospitalized SARS-CoV-2 cases, with disease onset from May 28, 2021 to December 4, 2021 (Pre-Omicron) and from December 23, 2021 to January 9, 2022 (Omicron). Among adolescents, two mRNA vaccine doses were associated with an 85% (aOR = 0.15; 95% CI: [0.04, 0.53]; p<0.01) lower likelihood of hospitalization among SARS-CoV-2 cases caused by Omicron. Among children, one mRNA vaccine dose was associated with a 79% (aOR = 0.21; 95% CI: [0.03, 0.77]; p<0.05) lower likelihood of hospitalization among SARS-CoV-2 cases caused by Omicron. The calculation of E-values, which quantifies how strong an unmeasured confounder would need to be to nullify our findings, suggest that these effects are unlikely to be explained by unmeasured confounding.

**Conclusions:**

Despite immune evasion by SARS-CoV-2 variants, vaccination continues to be associated with a lower likelihood of hospitalization among adolescent and pediatric Omicron (B.1.1.529) SARS-CoV-2 cases, even when the vaccines do not prevent infection. Continued efforts are needed to increase vaccine uptake among adolescent and pediatric populations.

## Introduction

Severe Acute Respiratory Syndrome (SARS-CoV-2) has caused more than 6.5 million deaths globally [1]. Safe and effective vaccines to prevent SARS-CoV-2 infection and severe outcomes have been approved since late 2020 for adults [2]. With the emergence of viral variants B.1.617.2 (Delta) in May 2021 and B.1.1.529 (Omicron) in late November 2021, decreased vaccine effectiveness against infection was observed [3–6]. Given that the goal of vaccination is to prevent death, severe disease, and overall disease burden, it is important to consider how well vaccines achieve these goals among individuals infected with SARS-CoV-2 [7].

Four recent studies focused on quantifying the real-world effectiveness of SARS-CoV-2 vaccination against hospitalization among adolescent or pediatric populations [8–11]. All the studies used uninfected controls, and resulting vaccine effectiveness estimates reflect the joint risk of SARS-CoV-2 infection and the risk of hospitalization conditional on infection [12]. Olson et al. [8] showed that BNT162b2 was 94% effective against hospitalization among adolescents infected with the Delta variant (B.1.617.2). After the emergence of the Omicron variant (B.1.1.529), two dose vaccine effectiveness against hospitalization was estimated to be 73% among adolescents ages 12 to 17 and 48% among pediatric patients ages 5 to 11 [9]. In a recent Morbidity and Mortality Weekly Report (MMWR), two dose vaccine effectiveness against hospitalization was between 73% and 94% among adolescent and pediatric populations [10]. In a study among hospitalized patients, two dose vaccine effectiveness against SARS-CoV-2 hospitalization was 93% during the Delta-dominant period and 40% during the Omicron-dominant period among adolescents, and 68% during the Omicron-dominant period among pediatric patients [11]. In contrast to the approach used in these studies, we conditioned upon SARS-CoV-2 infection in our analysis to estimate the direct association between vaccination and hospitalization risk among pediatric and adolescent SARS-CoV-2 cases [13].

The Canadian province of Ontario is a large (population 14.6 million) and diverse jurisdiction with high levels of SARS-CoV-2 vaccine coverage [14]. Approximately 87% of Ontario residents ages 12 to 17 and 53% of Ontario residents ages 5 to 11 received at least one SARS-CoV-2 dose as of January 2022 [15]. Most individuals ages 12–17 were eligible to receive the BNT162b2 (Pfizer-BioNTech, Comirnaty) vaccine beginning on May 28, 2021 [16], followed by ages 5 to 11 on November 28, 2021 [17]. Health Canada authorized Moderna Spikevax on August 27, 2021 for ages 12 to 17, but it was not authorized for ages 6 to 11 until after our study period [18]. We calculated one dose and two dose mRNA vaccine effectiveness against hospitalization among adolescent and pediatric SARS-CoV-2 cases to examine if vaccines prevent hospitalization when they fail to prevent SARS-CoV-2 infection.

## Methods

### Data

Robust public health surveillance systems in Ontario enable individual-level linkage of the SARS-CoV-2 vaccination database and the reportable disease database. Confirmed SARS-CoV-2 cases were identified in Ontario’s Public Health Case and Contact Management Solution (CCM). The CCM includes patient demographics (i.e., sex, age, comorbidities), geographic location (i.e., health region [5 units at the sub-provincial level]), and case characteristics (i.e., test date, symptom onset date, hospital admission and discharge dates) for all laboratory-confirmed SARS-CoV-2 cases in Ontario. SARS-CoV-2 vaccination information was identified from the provincial COVaxON database. COVaxON data includes vaccine administration information (i.e., dose dates, dose locations, dose indication, vaccine product) for Ontario residents with a provincial health insurance number. We linked data from CCM and COVaxON using a unique pseudo identifier present in both datasets. Median household income and percent visible minority at the census subdivision level were from the 2016 Census of Population [19].

### Study design

We conducted a retrospective cohort study of individuals aged 4–17 years who had a confirmed SARS-CoV-2 infection in Ontario, Canada. These individuals had a positive reverse transcription real-time polymerase chain reaction (PCR) test between May 28, 2021 and December 4, 2021 (pre-Omicron), or between December 23, 2021 and January 9, 2022 (Omicron). Our study period ended because Ontario restricted publicly funded PCR testing to only select members of the population [20].

Our analyses only included incident SARS-CoV-2 infections (re-infections were excluded). We excluded SARS-CoV-2 cases where individuals had received a vaccine not approved for use in this population in Canada (e.g., Johnson & Johnson/Janssen COVID-19 vaccine, Oxford-AstraZeneca COVID-19 vaccine), or had received three vaccine doses (ages 12–17) or two vaccine doses (ages 4–11). During the study period, adolescents were not eligible for third doses (i.e., booster dose), and the recommended interval between first and second doses for children was eight weeks [21]. We extracted the data on January 19, 2022, but only included test dates up to January 9, 2022, to account for delays between testing, hospitalization, and reporting. SARS-CoV-2 cases aged 4 years were included in the analysis because cases among pediatric patients aged 4 and aged 5 were in the same 2-year age group in these data, and pediatric patients who turned 5 in the 2021 calendar year were eligible for vaccination.

### Measures

The outcome was hospitalization due to SARS-CoV-2. Hospitalizations were identified by a reported hospital admission date, or a reported hospitalization or ICU admission due to SARS-CoV-2 in the CCM. The exposure was SARS-CoV-2 vaccination. We considered individuals one-dose vaccinated 14 or more days after the date the first vaccine dose was administered; individuals were considered two-dose vaccinated 14 or more days after the date the second vaccine dose was administered. Individuals infected with SARS-CoV-2 within 13 days of being vaccinated were excluded from our analyses.

Case onset date, age, sex, asthma, immunocompromising condition, and health region were included as confounders [11, 22]. A Directed Acyclic Graph (DAG) outlining the main hypothesized relationships between the variables is presented in **S1 Fig** [23]. Case onset date was the date of symptom onset for symptomatic cases and the specimen collection date for asymptomatic cases. In the CCM, age was reported in 2-year age groups, and asthma and immunocompromising condition were reported by the patient or provider. Health region included northern, eastern, central, or western Ontario, or Toronto [24]. Median household income and percent visible minority in 2016 at the census subdivision level were included as confounders in a sensitivity analysis.

### Statistical analysis

Standardized differences (SD) [25, 26] and statistical tests (Fisher’s exact tests and unpaired t-tests) were used to compare differences in baseline characteristics between hospitalized and non-hospitalized cases. Standardized mean differences are not impacted by sample size, in contrast to the common statistical tests used in descriptive tables. A standardized difference greater than 0.10 represents a lack of covariate balance [27].

We used multivariable logistic regression to calculate the association between vaccination and hospitalization among adolescent (ages 12–17) and pediatric (ages 4–11) SARS-CoV-2 cases, while adjusting for case onset date, age, sex, asthma, immunocompromising condition, and health region. We examined one-dose and two-dose vaccine effectiveness among the adolescent population prior to the emergence (May 28, 2021 to December 4, 2021) and after the emergence of Omicron (December 23, 2021 to January 9, 2022), and one-dose vaccine effectiveness among the pediatric population after the emergence of Omicron. Ontario residents under age 12 were ineligible for SARS-CoV-2 vaccination during most of the pre-Omicron time period. Dates were chosen to align with the rise in the prevalence of the Omicron variant in Ontario. In the pre-Omicron period, less than 10% of SARS-CoV-2 samples had S-gene target failure and in the latter period, more than 90% of SARS-CoV-2 samples had S-gene target failure [28]. Vaccine effectiveness (VE) was calculated using the formula VE = (1-aOR)*100%.

We performed additional analyses to quantify the impact of unmeasured confounding and to explore the robustness of our results to our assumptions. First, we calculated E-values based on the results of our multivariable logistic regression models [29]. An E-value quantifies how strong an unmeasured confounder would need to be to explain away the association between SARS-CoV-2 vaccination and hospitalization, conditional on other measured covariates [30]. We calculated an E-values using the following formula [29]: E-value = $OR+\sqrt{OR*(1-OR)}$. Although there is no accepted threshold in the literature for an E-value that demonstrates robustness to unmeasured confounding [31], an E-value is a useful measure to quantify the strength of unmeasured confounding needed to alter our findings [32]. We conducted four model-based sensitivity analyses. First, we allowed for a 14-day delay between case onset date and hospitalization, where SARS-CoV-2 cases with onset dates after January 5, 2022 were excluded. In our main analysis, we allowed for a 10-day delay between case onset date and hospitalization. Second, we repeated our analyses with adjustment for specimen collection date as opposed to case onset date to lessen the impact of differential case onset dates by symptom status. Third, we only included symptomatic individuals as opposed to all SARS-CoV-2 cases. Fourth, we examined the impact of including median household income and percent visible minority at the census subdivision level as confounders in addition to sex, asthma, immunocompromising condition, age, and case onset date in a multivariable logistic regression model. Individual-level measures of socioeconomic status and race were not available in our data and are therefore unmeasured confounders in our study [11]. This study is reported as per the Strengthening the Reporting of Observational Studies in Epidemiology (STROBE) guideline.

### Ethics

We received ethics approval for this study from the Research Ethics Board at the University of Toronto (#00031358). This study includes secondary analysis of public health surveillance data collected under Ontario’s Health Protection and Promotion Act. Therefore, the need for informed consent was waived by the ethics committee.

## Results

**Fig 1:**
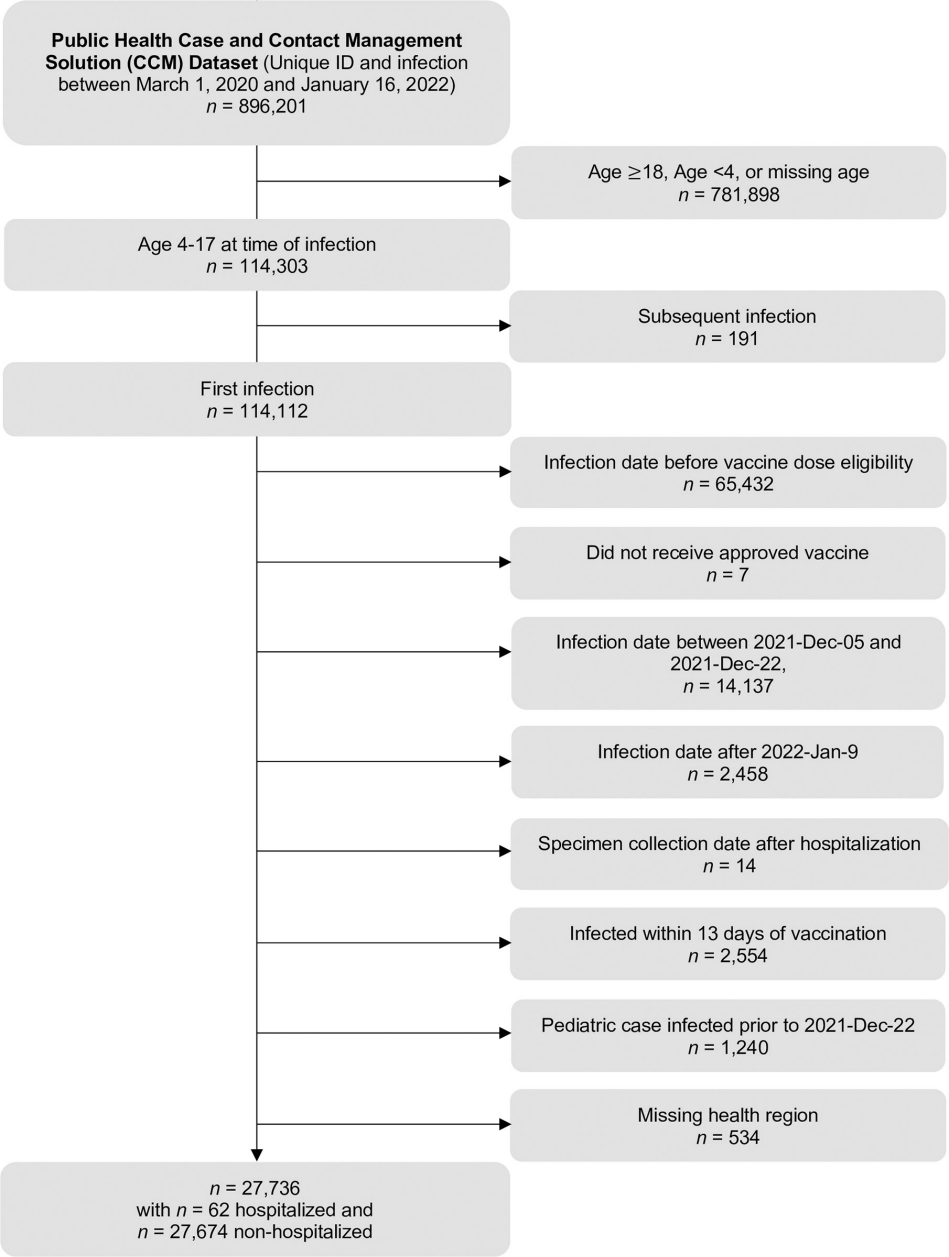
Flow diagram for creation of cohort.

In total, 62 hospitalized SARS-CoV-2 cases and 27,674 non-hospitalized SARS-CoV-2 cases were included (**Fig 1; Table 1**). In the full cohort across both age groups and the two time periods, individuals hospitalized with SARS-CoV-2 were more likely to be unvaccinated, immunocompromised, have an asthma diagnosis, live in the West or North health regions, and have an earlier case onset date (**Table 1**). We were unable to present frequencies, percentages, means, and standard deviations for the cohort stratified by age group and time period due to small cell sizes, however the relationships were quantified using standardized differences and statistical tests (**S1 Table**).

**Table 1:** Description of adolescent and pediatric SARS-CoV-2 cases by hospitalization status (*n* = 27,736).

Characteristic	Full Cohort					
	Hospitalized		Non-hospitalized		SD	*p* ^*a*^
	*n* = 62		*n* = 27,674			
	*n*	(%)	*n*	(%)		
Vaccination Status^*b*^					0.73	<0.001
Unvaccinated	47	(75.8)	11,716	(42.3)		
One dose	5	(8.1)	4,749	(17.2)		
Two doses	10	(16.1)	11,209	(40.5)		
Male					0.03	0.84
Yes	30	(48.4)	13,756	(49.7)		
No	32	(51.6)	13,918	(50.3)		
Age					0.30	0.51
4–5 years	5	(8.1)	2,100	(7.6)		
6–7 years	6	(9.7)	2,613	(9.4)		
8–9 years	5	(8.1)	3,006	(10.9)		
10–11 years	4	(6.5)	3,334	(12.0)		
12–13 years	10	(16.1)	4,715	(17.0)		
14–15 years	10	(16.1)	5,102	(18.4)		
16–17 years	22	(35.5)	6,804	(24.6)		
Immunocompromised					0.50	<0.001
Yes	7	(11.3)	40	(0.01)		
No	55	(88.7)	27,634	(99.9)		
Asthma					0.33	<0.001
Yes	4	(6.5)	143	(5.0)		
No	58	(93.5)	27,491	(99.5)		
Region					0.45	<0.01
Central	16	(25.8)	10,378	(37.5)		
East	16	(25.8)	6,134	(22.2)		
North	8	(12.9)	1,208	(4.4)		
Toronto	2	(3.2)	2,414	(8.7)		
West	20	(32.3)	7,540	(27.2)		
	**Mean**	**(Std)**	**Mean**	**(Std)**		
Case Onset Date^*c*^	2021-10-26	(82 days)	2021-12-10	(48 days)	0.66	<0.001

*Notes*: SD = standardized difference; Std = standard deviation

^*a*^ Fischer’s exact test for categorical variables, and unpaired t-tests for continuous variables

^*b*^ Vaccination status on case onset date

^*c*^ Date of symptom onset for symptomatic cases and the specimen collection date for asymptomatic cases

In the pre-Omicron period after adjustment for sex, asthma, immunocompromising condition, age, health region, and case onset date, we observed that one and two SARS-CoV-2 vaccine doses were not significantly associated (adjusted OR (aOR) one dose = 3.09 [95% CI: 0.31, 16.84], *p* = 0.26; aOR two doses = 0.90 [95% CI: 0.22, 2.86), *p* = 0.15) with additional protection against hospitalization among adolescent SARS-CoV-2 cases (**Table 2**). In the Omicron period after adjustment for sex, asthma, immunocompromising condition, age, health region, and case onset date, one SARS-CoV-2 dose was not significantly associated (aOR = 0.90 [95% CI: 0.05, 5.83], *p* = 0.93) with additional protection against hospitalization, but two SARS-CoV-2 doses were associated with an 85% (aOR = 0.15, [95% CI: 0.04, 0.53], *p*<0.01) lower likelihood of hospitalization among adolescent SARS-CoV-2 cases. After adjustment for sex, asthma, immunocompromising condition, age, health region, and case onset date, one SARS-CoV-2 vaccine dose was associated with a 79% (aOR = 0.21, [95% CI: 0.03, 0.77], *p*<0.05) lower likelihood of hospitalization among pediatric SARS-CoV-2 cases (ages 4–11) in the Omicron period (**Table 2**).

**Table 2:** Adjusted odds ratios of the association between vaccination status and hospitalization among adolescent and pediatric SARS-CoV-2 cases (*n* = 27,736).

Characteristic	Adolescent						Pediatric		
	Pre-Omicron			Omicron			Omicron		
	2021-May-28 to 2021-Dec-05 *n* = 4,999 (30 hospitalized)			2021-Dec-23 to 2022-Jan-09 *n* = 11,664 (12 hospitalized)			2021-Dec-23 to 2022-Jan-09 *n* = 11,073 (20 hospitalized)		
	aOR	(95% CI)	*p*	aOR	(95% CI)	*p*	aOR	(95% CI)	*p*
Vaccination Status^*b*^									
Unvaccinated	1.00	(ref)		1.00	(ref)		1.00	(ref)	
One dose	3.09	(0.31, 16.84)	0.26	0.90	(0.05, 5.83)	0.93	0.21	(0.03, 0.77)	<0.05
Two doses	0.90	(0.22, 2.86)	0.87	0.15	(0.04, 0.53)	<0.01	--	--	
Male									
Yes	0.81	(0.37, 1.71)	0.58	1.81	(0.57, 6.33)	0.32	0.71	(0.28, 1.75)	0.45
No	1.00	(ref)		1.00	(ref)		1.00	(ref)	
Age^*c*^	1.36	(1.07, 1.78)	<0.05	0.94	(0.66, 1.35)	0.74	0.92	(0.75, 1.13)	0.43
Immunocompromised									
Yes	126.16	(32.05, 462.37)	<0.001	--^*a*^	--		107.91	(14.52, 528.88)	<0.001
No	1.00	(ref)		1.00	(ref)		1.00	(ref)	
Asthma									
Yes	4.24	(0.67, 15.03)	0.06	40.22	(1.90, 295.74)	<0.01	28.65	(1.44, 177.65)	<0.01
No	1.00	(ref)		1.00	(ref)		1.00	(ref)	
Region									
Central	1.00	(ref)		1.00	(ref)		1.00	(ref)	
East	1.05	(0.30, 3.30)	0.93	4.64	(1.03, 32.10)	0.06	1.54	(0.44, 5.21)	0.48
North	2.74	(0.71, 9.12)	0.11	7.56	(0.89, 64.23)	<0.05 ^*d*^	3.32	(0.46, 15.40)	0.16
Toronto	0.25	(0.01, 1.76)	0.24	--^*a*^	--		0.75	(0.04, 4.54)	0.79
West	1.11	(0.44, 2.98)	0.82	1.36	(0.16, 11.49)	0.76	1.75	(0.53, 5.76)	0.34
Case Onset Date^*d*^	0.99	(0.98, 1.00)	<0.01	1.14	(1.00, 1.29)	<0.05	1.22	(1.11, 1.36)	<0.001

^*a*^ No individuals who were immunocompromised or in the Toronto health region were included in this analysis due to too few hospitalized cases with these characteristics

^*b*^ Vaccination status on case onset date

^*c*^ Age as continuous

^*d*^ Date of symptom onset for symptomatic cases and the specimen collection date for asymptomatic cases; ^*d*^ Discrepancy in significance is due to comparing a likelihood-based 95% CI and a Wald *p*-value

The E-value for the adjusted association between vaccination with two doses and the risk of hospitalization in our primary analysis among the adolescent population in the Omicron period (i.e., aOR = 0.15) was 12.8 [29]. An unmeasured confounder would need to be independently associated with vaccination and hospitalization by a 12.8-fold risk ratio to result in a null association, after adjusting for sex, asthma, immunocompromising condition, age, health region, and case onset date. Similarly, the E-value for the upper 95% CI bound (i.e., 95% CI upper bound = 0.53) is 3.2. A set of unmeasured confounders would need to be independently associated with vaccination and hospitalization by a 3.2-fold risk ratio for the 95% CI to encompass the null value after controlling sex, asthma, immunocompromising condition, age, health region, and case onset date. The E-value for the adjusted association between vaccination with one dose and the risk of hospitalization among the pediatric population (i.e., aOR = 0.21) was 9.0 and the E-value for the upper 95% CI bound (i.e., 95% CI upper bound = 0.77) was 1.9.

Our vaccine effectiveness estimates did not substantially change when we matched on specimen collection date, when we only included symptomatic cases, and when we included income and visible minority status at the census subdivision level (**S2 Table**). In our sensitivity analysis where only cases with onset dates prior to January 5, 2022 were included, the association between one vaccine dose and hospitalization among pediatric Omicron SARS-CoV-2 cases was invalid (due to too few hospitalizations in the time period), but the association among adolescent Omicron SARS-CoV-2 cases remained unchanged compared to our main analysis (**S2 Table**).

## Discussion

In this population-based analysis from the Canadian province of Ontario, we find evidence that mRNA vaccines against SARS-CoV-2 prevent hospitalization when adolescents receive a full two dose series and children received one dose in the context of an epidemic dominated by the immune-evasive Omicron variants. Among adolescent SARS-CoV-2 cases one vaccine dose was not significantly associated with a lower likelihood of protection against hospitalization in the pre-Omicron and Omicron periods, and two doses were not significantly protective in the pre-Omicron period. Our vaccine effectiveness estimates demonstrate robustness to unmeasured confounding, as evidenced by the high E-values for the estimates. As all subjects in this study had SARS-CoV-2 infection, the protection afforded by vaccination against hospitalization relates to attenuation of disease and is independent of protection provided against infection. This may in part explain our finding that vaccination was ineffective at preventing hospitalization in adolescents conditional upon infection with the Delta variant. SARS-CoV-2 infection with the Delta variant was less common among vaccinated individuals, and our study cohort during the Delta time period likely had a higher proportion of high risk population members compared to during the Omicron period [33].

The studies that have focused on SARS-CoV-2 vaccine effectiveness against hospitalization in adolescent and pediatric populations are not directly comparable to ours due to differences in control selection. Our estimated vaccine effectiveness estimates against hospitalization are lower than most estimates from studies using negative controls in the United States [8–10], likely because we isolated the impact of vaccination of hospitalization risk, independent of susceptibility to SARS-CoV-2 infection. In a case-control study conducted between May and October 2021 (i.e., prior to widespread infection with Omicron) with test-negative and syndrome-negative controls, vaccine effectiveness against hospitalization was 94% among those with two doses and 97% among those with one dose [8]. A study with 164 hospitals in the United States from April 2021 to January 2022 found that two dose vaccine effectiveness against hospitalization was 74% among children ages 5–11 years and between 73% and 94% among adolescents [10].

In contrast, two dose vaccine effectiveness was calculated to be 40% against hospitalization among adolescents during the circulation of Omicron in a study including 31 hospitals across the United States [11]. This lower estimate but may be due to earlier adolescent vaccination dates in the United States compared to in Ontario, as well as the impact of vaccine waning [11]. Unlike prior studies, our control group consisted of non-hospitalized SARS-CoV-2 cases, not uninfected individuals. This allowed for the isolation of the effectiveness of vaccination against hospitalization independent of infection risk, which explains our lower vaccine effectiveness estimates against hospitalization compared to the majority of prior studies [12].

Our study has several notable strengths. Due to Ontario’s high quality public health surveillance data, we isolated the impact of vaccination on hospitalization risk with control for individual level demographic and health related factors. Our study includes the population of a diverse region with publicly funded healthcare [34, 35]. We used a quantitative bias analysis to quantify the susceptibility of our results to unmeasured confounding [29]. Our study also had a few limitations. First, we did not consider time since vaccination in our analysis. In a recent study in the United States, time since vaccination was not shown to significantly impact SARS-CoV-2 vaccine effectiveness estimates against hospitalization in younger individuals [10]. We were unable to assess two dose effectiveness against hospitalization specifically among cases ages 4–11 years due to few hospitalizations among two-dose vaccinated individuals. Additionally, with only 62 total hospitalizations included in our study, we were likely underpowered to detect all true vaccine effects. Vaccination may be protective where our study showed a lack of statistical significance (i.e., Type II error), and a larger study over a longer period is needed. Some individuals aged four in our study were ineligible for vaccination. If they had a higher incidence of hospitalization, our vaccine effectiveness estimates would be biased away from the null. The availability of testing, and the propensity to get tested may have differed between vaccinated and unvaccinated individuals, and the comorbidities may have been more completely ascertained among hospitalized SARS-CoV-2 cases. Finally, comorbidities (i.e., asthma and immunocompromising conditions) in the CCM did not have a standardized definition which leads to residual confounding.

With the continued emergence of variants that may further decrease SARS-CoV-2 vaccine effectiveness against infection, it is vital to consider how effective these vaccines are at preventing severe outcomes [36]. Future studies should examine the impact of subsequent vaccine doses in adolescent and pediatric populations on SARS-CoV-2 hospitalization risk with emerging variants.

## Conclusions

In this evaluation of the effectiveness of SARS-CoV-2 vaccination in adolescent and pediatric Omicron cases ages 4 to 17 in Ontario, Canada, we found that vaccination with two doses among adolescents and one dose among children is associated with a decreased likelihood of hospitalization, even when the vaccines do not prevent infection. SARS-CoV-2 vaccines remain an effective intervention to prevent severe outcomes in adolescent and pediatric populations, and continued efforts are needed to increase vaccine uptake in these populations.

## Supporting information

S1 Fig Directed Acyclic Graph (DAG). DAG of the relationship between vaccination status and hospitalization among adolescent and pediatric SARS-CoV-2 cases.
(TIF)

S1 Table Description of adolescent and pediatric SARS-CoV-2 cases by hospitalization status, stratified by age and time period (*n* = 27,736). (DOCX)

S2 Table Adjusted odds ratios of the association between vaccination status and hospitalization among adolescent and pediatric SARS-CoV-2 cases with varying assumptions. (DOCX)
