# Supplementary figures and images for: Vaccine effectiveness against hospitalization among adolescent and pediatric SARS-CoV-2 cases between May 2021 and January 2022 in Ontario, Canada: A retrospective cohort study
Source: PLoS One. 2023 Mar 31;18(3):e0283715. doi: 10.1371/journal.pone.0283715 (PMC10065234; doi:10.1371/journal.pone.0283715)

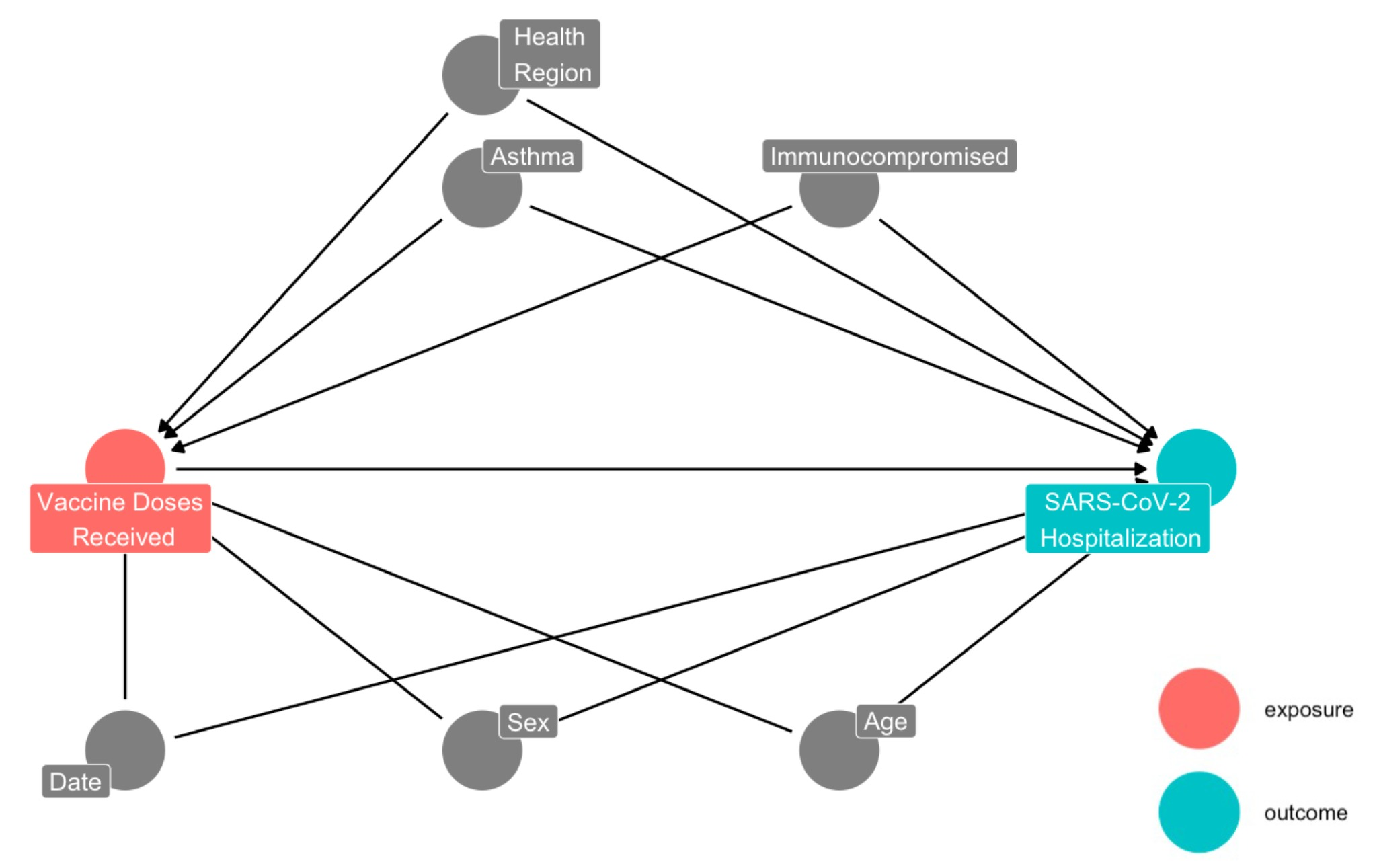

Supplement: S1 Fig — DAG of the relationship between vaccination status and hospitalization among adolescent and pediatric SARS-CoV-2 cases. (TIF) [file pone.0283715.s001.tif]
