# Supplementary material for: Vaccine effectiveness against hospitalization among adolescent and pediatric SARS-CoV-2 cases between May 2021 and January 2022 in Ontario, Canada: A retrospective cohort study
Source: PLoS One. 2023 Mar 31;18(3):e0283715. doi: 10.1371/journal.pone.0283715 (PMC10065234; doi:10.1371/journal.pone.0283715)
Supplement: S1 Table — (DOCX) [file pone.0283715.s002.docx]

| Characteristic | Adolescent | | | | Pediatric | |
| --- | --- | --- | --- | --- | --- | --- |
|  | **Pre-Omicron**  2021-May-28 to 2021-Dec-05  *n* = 4,999 (30 hospitalized) | | **Omicron**  2021-Dec-23 to 2022-Jan-09  *n* = 11,664 (12 hospitalized) | | **Omicron**  2021-Dec-23 to 2022-Jan-09  *n* = 11,073 (20 hospitalized) | |
|  | **SD** | ***p****^a^* | **SD** | ***p****^a^* | **SD** | ***p****^a^* |
| Vaccination Status*^b^* | 0.28 | 0.11 | 0.92 | <0.01 | 0.75 | <0.01 |
| Male | 0.08 | 0.81 | 0.22 | 0.63 | 0.14 | 0.69 |
| Immunocompromised | 0.62 | <0.001 | 0.05 | 1.00 | 0.46 | <0.001 |
| Asthma | 0.22 | 0.28 | 0.42 | <0.001 | 0.30 | <0.05 |
| Region | 0.28 | 0.50 | 0.95 | 0.03 | 0.37 | 0.54 |
| Age*^c^* | 0.49 | <0.05 | 0.40 | 0.40 | 0.28 | 0.70 |
| Case Onset Date*^d^* | 0.48 | <0.01 | 0.59 | <0.05 | 0.88 | <0.001 |

*Notes:* SD = standardized mean difference

*^a^* Fisher’s exact test for categorical variables, and unpaired t-tests for continuous variables; *^b^* Vaccination status on case onset date; *^c^* Age in categories;

*^d^* Date of symptom onset for symptomatic cases and the specimen collection date for asymptomatic cases
