# Supplementary material for: Vaccine effectiveness against hospitalization among adolescent and pediatric SARS-CoV-2 cases between May 2021 and January 2022 in Ontario, Canada: A retrospective cohort study
Source: PLoS One. 2023 Mar 31;18(3):e0283715. doi: 10.1371/journal.pone.0283715 (PMC10065234; doi:10.1371/journal.pone.0283715)
Supplement: S2 Table — (DOCX) [file pone.0283715.s003.docx]

| Vaccination Status*^a^* | Adolescent | | | | | | Pediatric | | |
| --- | --- | --- | --- | --- | --- | --- | --- | --- | --- |
|  | **Pre-Omicron**  2021-May-28 to 2021-Dec-05 | | | **Omicron**  2021-Dec-23 to 2022-Jan-09 | | | **Omicron**  2021-Dec-23 to 2022-Jan-09 | | |
|  | **aOR** | **(95% CI)** | ***p*** | **aOR** | **(95% CI)** | ***p*** | **aOR** | **(95% CI)** | ***p*** |
| Model 1: SARS-CoV-2 cases hospitalized after January 5, 2022 were excluded | | | | | | | | | |
| Unvaccinated  One dose  Two doses | -- | -- |  | 1.00  0.93  0.10 | *n* = 10,727  (ref)  (0.05, 6.14)  (0.02, 0.39) | 0.95  <0.001 | --*^b^* | -- |  |
| Model 2: Adjusted logistic regression model included specimen collection date as a covariate as opposed to case onset date | | | | | | | | | |
| Unvaccinated  One dose  Two doses | 1.00  3.27  0.89 | *n* = 4,999  (ref)  (0.32, 18.10)  (0.22, 2.84) | 0.24  0.86 | 1.00  0.88  0.15 | *n* = 11,664  (ref)  (0.05, 5.71)  (0.04, 0.52) | 0.91  <0.05 | 1.00  0.20  -- | *n* = 11,073  (ref)  (0.03, 0.74)  -- | <0.05 |
| Model 3: Only symptomatic SARS-CoV-2 cases on the date of specimen collection were included | | | | | | | | | |
| Unvaccinated  One dose  Two doses | 1.00  3.11  0.25 | *n* = 3,893  (ref)  (0.24, 20.64)  (0.01, 1.56) | 0.31  0.22 | 1.00  2.44  0.11 | *n* = 3,062  (ref)  (0.11, 24.29)  (0.02, 0.61) | 0.48  <0.01 | 1.00  0.16  -- | *n* = 3,106  (ref)  (0.01, 0.87)  -- | 0.08*^c^* |
| Model 4: Income and percent visible minority at the census subdivision level were included as additional covariates | | | | | | | | | |
| Unvaccinated  One dose  Two doses | 1.00  2.95  0.94 | *n* = 4,999  (ref)  (0.30, 15.96)  (0.24, 2.98) | 0.28  0.92 | 1.00  0.92  0.14 | *n* = 11,664  (ref)  (0.05, 5.97)  (0.03, 0.51) | 0.94  <0.01 | 1.00  0.23  -- | *n* = 11,073  (ref)  (0.04, 0.83)  -- | 0.05 |

*Note:* All models are adjusted for sex, age, immunocompromising condition, asthma, region, and case onset date, unless otherwise specified.

*^a^* Vaccination status on case onset date; *^b^* There were too few hospitalized cases in the pediatric omicron period prior to January 5, 2022, and thus this population was excluded from the analysis; *^c^* Discrepancy in significance is due to comparing a likelihood-based 95% CI and a Wald *p*-value
